# Comparison of 10 obesity-related indices for predicting hypertension based on ROC analysis in Chinese adults

**DOI:** 10.3389/fpubh.2022.1042236

**Published:** 2022-11-25

**Authors:** Xiaohan Lee, Yanan Gao, Yuting Zhang, Yong Feng, Linna Gao, Aiwen Wang, Yongbao Jiang, Huiming Huang

**Affiliations:** ^1^Faculty of Sports Science, Ningbo University, Ningbo, China; ^2^Research Academy of Grand Health, Ningbo University, Ningbo, China; ^3^Affiliated Hospital of Ningbo University, Ningbo, China

**Keywords:** hypertension, general obesity-related indices, central obesity-related indices, predictive performance, ROC analysis

## Abstract

**Objective:**

To compare the predictive performance of the percentage body fat (PBF), body mass index (BMI), waist circumference (WC), hip circumference (HC), waist–hip ratio (WHR), waist–height ratio (WHtR), a body shape index (ABSI), body roundness index (BRI), abdominal volume index (AVI), and conicity index (CI) for identifying hypertension.

**Methods:**

A cross-sectional study was conducted among 2,801 adults (1,499 men and 1,302 women) aged 18 to 81 in Ningbo, China. The receiver operator characteristic (ROC) analysis and multiple non-parametric Z tests were used to compare the areas under the curve (AUC). The maximum Youden's indices were used to determine the optimal cut-off points of 10 obesity-related indices (ORI) for hypertension risk.

**Results:**

The AUC of all the indices were statistically significant (*P* < 0.05). The AUC of all the indices in men and women were 0.67–0.73 and 0.72–0.79, respectively. Further non-parametric Z tests showed that WHR had the highest AUC values in both men [0.73 (95% CI: 0.70, 0.76)] and women (0.79 (95% CI: 0.75, 0.83)], and several central ORI (men: WHR, WC, BRI, AVI, and CI, 0.71–0.73; women: WC, WHR, and AVI, 0.77–0.79) were higher than general ORI (PBF and BMI, 0.68 in men; 0.72–0.75 in women), with adjusted *P* < 0.05. The optimal cut-off points for identifying hypertension in men and women were as follows: PBF (23.55%, 32.55%), BMI (25.72 kg/m^2^, 23.46 kg/m^2^), HC (97.59 cm, 94.82 cm), WC (90.26 cm, 82.78 cm), WHR (0.91, 0.88), WHtR (0.51, 0.55), ABSI (0.08 m^7/6^/kg^2/3^, 0.08 m^7/6^/kg^2/3^), BRI (4.05, 4.32), AVI (16.31 cm^2^, 13.83 cm^2^), and CI (1.23 m^2/3^/kg^1/2^, 1.27 m^2/3^/kg^1/2^). Multivariate logistic regression models showed that all indices were statistically significant (*P* < 0.05) with the adjusted ORs (per 1-SD increase) at 1.39–2.06 and ORs (over the optimal cut-off points) at 1.80–2.64.

**Conclusions:**

All 10 ORI (PBF, BMI, HC, WC, WHR, WHtR, ABSI, BRI, AVI, and CI) can effectively predict hypertension, among which WHR should be recommended as the best predictor. Central ORI (WHR, WC, and AVI) had a better predictive performance than general ORIs (PBF and BMI) when predicting the risk of hypertension.

## Introduction

Hypertension is a risk factor for cardiovascular diseases (CVD) and death; thus, it is an essential global health threat (1, 2). A nationwide epidemiological study involving 451,755 residents from 31 provinces in China showed that the prevalence of hypertension among adults in China was 23.2% (3). By 2025, the number of adults with hypertension is expected to reach 1.56 billion (4). Therefore, preventing and reducing the incidence of hypertension has become an urgent problem. Some research have revealed that obesity is a significant, independent, and modifiable risk factor for hypertension and other CVD (5, 6). Obesity-related indices (ORI) have become the primary choice for health risk screening because of their convenience and economy (7).

Speculation has grown in recent years about which measure of obesity is a better predictor of hypertension. However, there are some contradictions in some studies. Body mass index (BMI) is commonly used in many obesity studies. However, it was reported in many studies that it failed to distinguish between body fat and lean body mass (8, 9). Several central obesity indices, such as waist–hip ratio (WHR), waist circumference (WC), and waist–height ratio (WHtR), were considered better indices of CVD risk because they reflect body fat distribution and upper body adiposity. Some studies indicated that WC is the best indicator for reflecting the associations between obesity and hypertension risk (10, 11). However, WC does not account for differences in height, so risk assessments for tall and short people may be too high and too low, respectively. Jensen et al.'s and Calderón-García et al.'s views were that measuring WHR is not advantageous over measuring WC alone and is not recommended as part of routine obesity assessment (12, 13). Several studies proposed the WHtR as an alternative to WC (5, 8). A meta-analysis concluded that WHtR was the best predictor of hypertension in both sexes (14). Moreover, several studies indicated that women's hip circumference (HC) is a more robust independent predictor of death and CVD development than BMI or WC (15, 16).

Researchers have explored several new ORI to improve the above limitations, such as a body shape index (ABSI), body roundness index (BRI), visceral adiposity index (VAI), Chinese visceral adiposity index (CVAI), lipid accumulation product (LAP), abdominal volume index (AVI), and conicity index (CI). The ABSI was developed in 2012 by Krakauer et al., combining BMI, WC, and height (17). A high ABSI relates to a greater fraction of abdominal adipose tissue and appears to be a significant risk factor for premature death (17). In 2013, Thomas et al. developed the BRI, which is a new geometrical index that combines height and WC to predict the percentage body fat (PBF) and evaluate health status (18). Amato et al. set up a VAI in 2010 that integrated WC, triglyceride (TG), high-density lipoprotein cholesterol (HDL–C), and BMI (19). Visceral obesity in Chinese can be assessed with CVAI, which is developed by combining BMI, age, WC, TG, and HDL–C (20). The LAP is based on combining two measurements (WC and TG) (21). Although many studies have verified that VAI, CVAI, and LAP were reliable visceral adiposity measures (19–22), their calculation requires biochemical blood indices (HDL–C and TG), which are not easy to measure. The AVI is another anthropometric tool for assessing the whole volume. In addition, CI is often used in epidemiological studies. However, it is not known whether the new ORI is a better predictor of hypertension presence or risk than the traditional ORI for Chinese.

PBF reflects body composition more accurately than traditional ORI. Recent studies demonstrated that PBF is a risk factor for CVD, including hypertension (23). The fifth physical monitoring of the General Administration of Sport first adopted the bioelectrical impedance method to measure PBF on a large scale in China in 2020 (24). However, the measurement of PBF is more complex than traditional ORI, and many studies have not included PBF (8, 25, 26). Therefore, the predictive performance of PBF compared with traditional ORI for identifying hypertension among the same large population still remained unclear.

Previous studies showed that the hypertension prevalence and the ORI values indicate significant differences with sex (4, 5, 27–30). Therefore, we conducted a cross-sectional study in both sexes to (1) compare the predictive performance of 10 easy-to-measure ORI (PBF, BMI, HC, WC, WHR, WHtR, ABSI, BRI, AVI, and CI) for identifying hypertension except the indices that needed biochemical blood indices (such as VAI, CVAI, and LAP); and (2) determine the optimal cut-off points for 10 types of ORI to predict hypertension.

## Materials and methods

### Study design

This study was designed as a cross-sectional study. The study methods complied with the Strengthening the Reporting of Observational Studies in Epidemiology (STROBE) Statement.

### Participants

We recruited participants who received a routine physical fitness examination at the Affiliated Hospital of Ningbo University from 2018 to 2019 in Ningbo City. Participants were recruited by inclusion and exclusion criteria. Inclusion criteria: (1) age from 18 to 81 years old; (2) volunteered for this study; and (3) independent completion of the body composition test, medical examination, and physical fitness test). Exclusion criteria (1) pregnant, using a pacemaker, wheelchair-bound, unable to stand, an amputee, unable to grip the handles of the analyzer, or unwilling to take off their shoes for body composition test; (2) severe CVD and infectious disease; and (3) inability to complete the physical fitness examination. Initially 2,813 participants were recruited. A total of 12 participants were excluded because the body composition test data were missing. The final sample size was 2801 participants, comprising 1,499 men and 1,302 women. Informed consent in a signed form was obtained from involved participants. The design and protocol of this study were approved by the Institutional Review Board of the Faculty of Sports Science, Ningbo University (NO. 2018RAGH1025).

### Measurements and definitions

#### Dependent variables

The dependent variable in this study was blood pressure. The participants were advised to avoid caffeinated beverages and exercise for at least 30 min before the measurement (31). Each participant's seated brachial blood pressure was measured after at least 5 min of rest by a standardized automatic electronic sphygmomanometer (HEM-907; Omron, Kyoto, Japan). During the measurement, each participant was seated with their tested arm supported at the level of the heart. Systolic blood pressure (SBP) and diastolic blood pressure (DBP) were measured three times with 2-min intervals, and SBP and DBP were estimated by the average of these three successive reading values (32). Hypertension was defined as elevated blood pressure (SBP ≥ 140 mmHg and/or DBP ≥ 90 mmHg) or the patient having undergone antihypertensive medication therapy (32).

#### Independent variables

The ten types of easy-to-measure ORI (PBF, BMI, HC, WC, WHR, WHtR, ABSI, BRI, AVI, and CI) were collected by trained technicians following standard procedures in the study as independent variables. All measurements were performed with the participants standing upright, with light clothing and without shoes. Weight and PBF were measured by a bioimpedance body composition analyzer (Inbody720, Inbody Co. Ltd., Seoul, Korea). Each participant's height was measured using a stadiometer. An anthropometric tape was used to measure HC and WC. HC was measured around the thighs at the height of the greater trochanter (33). The WC measurements were obtained at the end of normal expiration at the midpoint level between the lower end of the 12th rib and the upper end of the iliac crest (33). Weight, height, HC, and WC were measured to the nearest 0.1 kg and 0.1 cm. The formula of ORI is shown in Table 1.

**Table 1:** ORI (formula and reference).

**Indices**	**Formula**	**Reference**
PBF (%)	*Fat mass* (*kg*)/*Weight* (*kg*) × 100%	(34)
BMI (kg/m^2^)	*Weight* (*kg*)/(*Height*)^2^ (*m*)	(35)
HC (cm)	-	-
WC (cm)	-	-
WHR	*WC* (*cm*)/*HC* (*cm*)	(35)
WHtR	*WC* (*cm*)/*Height* (*cm*)	(35)
ABSI (m^7/6^/kg^2/3^)	*WC* (*m*)/[*BMI*^2/3^(*kg*/*m*^2^) × *Height*^1/2^(*m*)]	(17)
BRI	$364.2-365.5\;\times \;\sqrt{1-{\left(\frac{WC\;\left(m\right)\;/\;2\pi }{0.5\;\times Height\;\left(m\right)}\right)}^{2}}$	(18)
AVI (cm^2^)	[2 × *WC*^2^ (*cm*) + 0.7 × (*WC* − *HC*)^2^ (*cm*)]/1000	(36)
CI (m^2/3^/kg^1/2^)	0.109^−1^ × *WC* (*m*) × [*Weight* (*kg*)/*Height* (*m*)]^−1/2^	(37)

ORI, obesity–related indices; PBF, percentage body fat; BMI, body mass index; HC, hip circumference; WC, waist circumference; WHR, waist–hip ratio; WHtR, waist–height ratio; ABSI, a body shape index; BRI, body roundness index; AVI, abdominal volume index; CI, conicity index.

#### Covariates

Previous studies indicated that several variables, such as age, rest heart rate (HR), cardiorespiratory fitness (CRF), and brachial-ankle pulse wave velocity (baPWV), arteriosclerosis, and lifestyle (smoking and exercise status), which may be associated with both obesity and hypertension, were considered as potential confounders (5, 38, 39). These covariates were classified as continuous and categorical variables. In this study, the continuous variables comprised age, HR, cardiorespiratory fitness (CRF), and brachial–ankle pulse wave velocity (baPWV). Questionnaires collected participants' ages. CRF was determined using a submaximal VO_2_ max test conducted on a stationary bicycle in accordance with the Ekblom-Bak cycle ergometer test (40), presented as relative values in ml/kg/min and analyzed by metabolic equivalent values (METs). CRF was not tested in participants over 60 years of age. The HR and baPWV were measured by a VP-1000 automated arteriosclerosis analyzer (Colin Medical Technology Corp., Komaki, Japan). Participants rested in a supine position for at least 5 min, wrapping four cuffs around their upper arms and ankles. Then, the upper and lower extremity arteries were measured simultaneously by a non-invasive shock pressure wave graph (38).

Categorical variables comprised age grade, arteriosclerosis, and lifestyle (smoking and exercise status). In this study, participants' ages ranged from 18 to 81 years. They were divided into two groups according to age: 18–49 and 50–81. In regular clinical and epidemiological settings, baPWV is the most widely used measure of arteriosclerosis. Arteriosclerosis was defined by a baPWV ≥1400 cm/s (41). The smoking status was defined as smoking at least one cigarette per day continuously or cumulatively for 6 months (42). The exercise status was defined as at least 30 minutes of moderate-intensity exercise thrice per week (43).

### Statistical analysis

Statistical analysis was performed with the SPSS software (version 26.0). Continuous variables (age, height, weight, PBF, BMI, HC, WC, WHR, WHtR, ABSI, BRI, AVI, CI, HR, CRF, baPWV, SBP, and DBP) are presented as the mean ± standard deviation (SD). Data are presented as numbers (%) for categorical variables (age grade, arteriosclerosis, smoking, and exercise status). The predictive performance of 10 types of ORI for identifying hypertension was evaluated by receiver operating characteristic (ROC) analysis. A multiple nonparametric Z-test was used to compare differences between different areas under the curve (AUC) of the ROC curves. When the Z value is >1.96, the *P*-value of the AUC difference between the two ORI is < 0.05 (44, 45). Furthermore, we adjusted the *P*-value in multiple hypothesis testing to minimize type I errors by False Discovery Rate (FDR) (adjusted *P* = original P × n/rank, n is the multiple test times, and rank is the original *P*-value' rank) (46). The optimal cut-off points of ORI were determined by the maximum Youden's index (sensitivity + specificity –1).

A multivariable logistic regression model was used to analyze the association between ORI and hypertension in two conditions: per 1-SD increase and over the optimal cut-off points. The odds ratios (ORs) were standardized by transformed observations [(observation – mean)/SD] in the models. Potential confounding factors included age, HR, arteriosclerosis, lifestyle (smoking and exercise status), and CRF. When there were covariables with missing data, the continuous variables were ignored, and the categorical variables used dummy variables.

The sensitivity analysis was performed by re-running the ROC analysis by the alternative cut-off point of hypertension (SBP ≥ 135 mmHg and/or DBP ≥ 85 mmHg) (47, 48).

## Results

### Participants characteristics at baseline

Table 2 showed the characteristics of the participants. The study enrolled 2,801 participants (526 with hypertension and 2,275 without hypertension), comprising 1,499 men (374 with hypertension and 1,125 without hypertension) and 1,302 women (152 with hypertension and 1,150 without hypertension). The SBP and DBP of hypertensive participants were 143.06 (11.84) and 87.31 (7.76), respectively. The missing data for the covariates were as follows: CRF, 552 (participants over 60 years of age were not tested); smoking status, 180; exercise status, 165.

**Table 2:** Participants characteristics at baseline.

**Characteristic**	**Overall (*n =* 2801)**		**Men (*n =* 1499)**		**Women (*n =* 1302)**	
	**Hypertension (*n =* 526)**	**No hypertension (*n =* 2275)**	**Hypertension (*n =* 374)**	**No hypertension (*n =* 1125)**	**Hypertension (*n =* 152)**	**No hypertension** **(*n =* 1150)**
Age (years)	50.58 (9.82)	42.26 (10.83)	49.40 (10.00)	43.13 (10.85)	53.49 (8.73)	41.41 (10.74)
Height (cm)	165.63 (7.80)	164.59 (7.89)	169.01 (5.70)	170.29 (5.82)	157.32 (5.76)	159.03 (5.24)
Weight (kg)	73.11 (11.19)	64.67 (11.11)	76.49 (9.81)	71.50 (9.40)	64.77 (9.94)	57.98 (8.21)
PBF (%)	27.53 (5.96)	25.37 (6.29)	25.08 (4.28)	21.65 (4.98)	33.56 (5.19)	29.02 (5.20)
BMI (kg/m^2^)	26.57 (3.10)	23.76 (3.00)	26.73 (2.81)	24.63 (2.78)	26.16 (3.71)	22.91 (2.96)
HC (cm)	99.28 (5.55)	94.78 (5.26)	99.58 (5.23)	95.76 (5.31)	98.56 (6.24)	93.81 (5.03)
WC (cm)	92.56 (9.35)	83.24 (9.21)	92.93 (8.59)	85.43 (9.06)	91.67 (10.99)	81.10 (8.86)
WHR	0.93 (0.04)	0.87 (0.05)	0.93 (0.04)	0.88 (0.04)	0.92 (0.58)	0.86 (0.05)
WHtR	0.54 (0.05)	0.50 (0.05)	0.53 (0.05)	0.48 (0.05)	0.57 (0.06)	0.51 (0.05)
ABSI (m^7/6^/kg^2/3^)	0.08 (0.003)	0.07 (0.003)	0.08 (0.003)	0.07 (0.004)	0.08 (0.004)	0.08 (0.003)
BRI	4.62 (1.34)	3.54 (1.12)	4.40 (1.09)	3.46 (1.07)	5.17 (1.70)	3.62 (1.17)
AVI (cm^2^)	17.35 (3.52)	14.13 (3.07)	17.46 (3.19)	14.84 (3.06)	17.10 (4.21)	13.44 (2.93)
CI (m^2/3^/kg^1/2^)	1.28 (0.07)	1.22 (0.07)	1.26 (0.06)	1.20 (0.07)	1.31 (0.07)	1.23 (0.06)
HR (beats/min)	73.24 (10.60)	71.69 (9.43)	73.78 (10.53)	71.91 (9.90)	71.98 (10.72)	71.49 (8.95)
CRF (Mets)	9.59 (2.60)	9.58 (2.17)	10.03 (2.51)	10.70 (2.04)	7.35 (1.86)	8.72 (1.84)
baPWV (cm/s)	1547.36 (233.43)	1234.08 (221.4)	1540.25 (230.92)	1292.34 (161.88)	1564.84 (239.35)	1177.10 (160.92)
SBP (mmHg)	143.06 (11.84)	114.26 (10.14)	141.67 (11.51)	118.04 (8.46)	146.48 (11.97)	110.56 (10.29)
DBP (mmHg)	87.31 (7.76)	68.53 (8.17)	87.87 (7.95)	71.89 (7.09)	85.92 (7.08)	65.25 (7.82)
Age grade, *n (%)*
18–49, *n (%)*	229 (43.54)	1671 (73.45)	175 (46.8)	803 (71.37)	54 (35.53)	868 (75.48)
50–81, *n (%)*	297 (56.46)	604 (36.55)	199 (53.2)	322 (28.62)	98 (64.47)	282 (24.52)
Arteriosclerosis, *n (%)*
No, *n (%)*	143 (27.19)	1917 (84.26)	106 (28.3)	871 (77.42)	37 (24.32)	1046 (90.96)
Yes, *n (%)*	383 (72.81)	358 (15.74)	268 (71.7)	254 (22.58)	115 (75.66)	104 (9.04)
Smoking status, *n (%)*
No, *n (%)*	311 (61.22)	1722 (81.50)	177 (49.0)	705 (67.85)	134 (91.16)	1017 (94.69)
Yes, *n (%)*	197 (38.78)	391 (18.50)	184 (51.0)	334 (32.15)	13 (8.84)	57 (5.31)
Exercise status, *n (%)*
No, *n (%)*	430 (83.33)	1683 (79.39)	308 (84.2)	855 (81.58)	122 (81.33)	828 (77.24)
Yes, *n (%)*	86 (16.67)	437 (20.61)	58 (15.8)	193 (18.42)	28 (18.67)	244 (22.76)

Values are expressed as mean (SD) or n (%). The missing values are as follows: CRF, 552 (not tested in those more than 60 years old); smoking status, 180; exercise status, 165. PBF, percentage body fat; BMI, body mass index; HC, hip circumference; WC, waist circumference; WHR, waist–hip ratio; WHtR, waist–height ratio; ABSI, a body shape index; BRI, body roundness index; AVI, abdominal volume index; CI, conicity index; HR, rest heart rate; CRF, cardiorespiratory fitness; Mets, metabolic equivalent; baPWV, brachial-ankle pulse wave velocity; SBP, systolic blood pressure; DBP, diastolic blood pressure; 10 types of ORI for predicting hypertension.

### Comparisons of ROC analyses of 10 ORI for predicting hypertension

**Figure 1:**
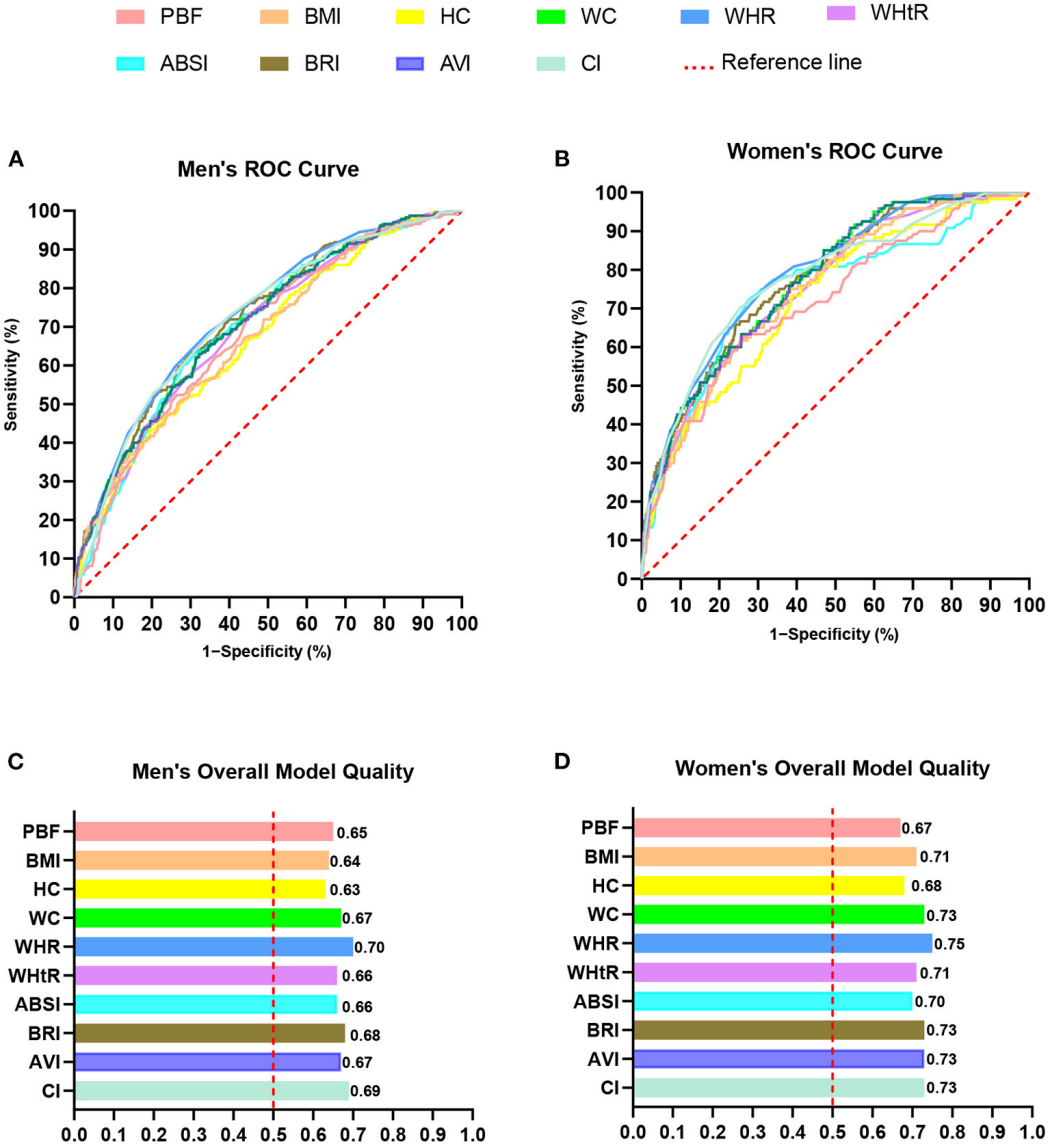
ROC analysis of ten types of ORI for predicting hypertension. **(A)** Men's ROC curve. **(B)** Women's ROC curve. **(C)** Men's overall model quality. **(D)** Women's overall model quality. ORI, obesity-related indices; PBF, percent body fat; BMI, body mass index; HC, hip circumference; WC, waist circumference; WHR, waist–hip ratio; WHtR, waist–height ratio; ABSI, a body shape index; BRI, body roundness index; AVI, abdominal volume index; CI, conicity index.

ROC analysis showed that the AUC of all the indices were statistically significant (*P* < 0.05). The AUC of all the indices in men and women were 0.67–0.73 and 0.72–0.79, respectively (Table 3). The ROC curve and overall model quality plot of 10 ORI for predicting hypertension by sex are shown in Figure 1. The ROC curves of all indices were above the reference line, indicating that the 10 types of ORI can be used to predict hypertension. The left side of a vertical red reference line in the overall model quality plot indicates that the lower limit of the 95% CI of AUC is < 0.5. As shown in Figure 1, the overall model quality plots show that the lower limit of the 95% CI of AUC of the 10 types of ORI is >0.5. The lower limit of the 95% CI of AUC (men, 0.70; women, 0.75) and AUC (men, 0.73, 95% CI: 0.70, 0.76; women, 0.79, 95% CI: 0.75, 0.83) of WHR was the largest. In men, the lower limit of the 95% CI of AUC (0.63) and AUC (0.67, 95% CI: 0.63, 0.71) of HC was the smallest. For women, the lower limit of the 95% CI of AUC (0.67) and AUC (0.72, 95% CI: 0.67, 0.77) of PBF was the smallest.

**Table 3:** The AUC of 10 types of ORI for predicting hypertension.

**Indices**	**Men (*n =* 1,499)**		**Women (*n =* 1,302)**	
	**AUC (95% CI)**	***P***	**AUC (95% CI)**	***P***
PBF (%)	0.68 (0.65, 0.72)	< 0.01	0.72 (0.67, 0.77)	< 0.01
BMI (kg/m^2^)	0.68 (0.64, 0.71)	< 0.01	0.75 (0.71, 0.80)	< 0.01
HC (cm)	0.67 (0.63, 0.71)	< 0.01	0.72 (0.68, 0.77)	< 0.01
WC (cm)	0.71 (0.67, 0.74)	< 0.01	0.77 (0.73, 0.82)	< 0.01
WHR	0.73 (0.70, 0.76)	< 0.01	0.79 (0.75, 0.83)	< 0.01
WHtR	0.70 (0.66, 0.73)	< 0.01	0.76 (0.71, 0.80)	< 0.01
ABSI (m^7/6^/kg^2/3^)	0.70 (0.67, 0.74)	< 0.01	0.75 (0.70, 0.80)	< 0.01
BRI	0.72 (0.68, 0.75)	< 0.01	0.78 (0.73, 0.82)	< 0.01
AVI (cm^2^)	0.71 (0.67, 0.74)	< 0.01	0.77 (0.73, 0.81)	< 0.01
CI (m^2/3^/kg^1/2^)	0.72 (0.69, 0.76)	< 0.01	0.78 (0.73, 0.82)	< 0.01

ORI, obesity–related indices; PBF, percentage body fat; BMI, body mass index; HC, hip circumference; WC, waist circumference; WHR, waist–hip ratio; WHtR, waist–height ratio; ABSI, a body shape index; BRI, body roundness index; AVI, abdominal volume index; CI, conicity index.

Table 4 showed the comparisons of AUC for predicting hypertension among 10 types of ORI by the multiple non-parametric Z test. Moreover, we used the FDR to adjust the *P*-value in multiple hypothesis testing to minimize type I errors. In men, the AUC of WHR was highest, and the AUC of PBF, BMI, and HC was lower (adjusted *P* < 0.05). The AUC of five central ORI (WHR, WC, BRI, AVI, and CI) were higher than general ORI (PBF and BMI), all with adjusted *P* < 0.05. Women showed a similar trend: the AUC of three central ORI (WHR, WC, and AVI) were higher than general ORI (PBF and BMI), all with adjusted *P* < 0.05. The AUC of WHR was the largest except for WC, AVI, and CI (adjusted *P* < 0.05). Moreover, the AUC of PBF and HC was < 5 central ORI (WC, WHR, BRI, AVI, and CI) in women (adjusted *P* < 0.05).

**Table 4:** Comparisons of AUC for predicting hypertension among 10 types of ORI by the multiple non-parametric Z tests.

**Comparator**	**Men (*n =* 1,499)**				**Women (*n =* 1,302)**			
	**AUC difference (95%CI)**	**Z**	***P***	**P^a^**	**AUC difference (95%CI)**	**Z**	***P***	**P^**a**^**
PBF—BMI	0.01 (−0.02, 0.03)	0.54	0.59	0.64	−0.03 (−0.06, −0.00)	−2.05	0.04	0.09
PBF—HC	0.02 (−0.01, −0.04)	1.17	0.24	0.29	−0.00 (−0.04, 0.03)	−0.23	0.82	0.88
PBF—WC	−0.02 (−0.04, −0.01)	−2.60	0.01	0.02	−0.05 (−0.08, −0.03)	−5.01	< 0.01	< 0.01
PBF—WHR	−0.05 (−0.06, −0.03)	−6.01	< 0.01	< 0.01	−0.07 (0.10, −0.05)	−5.67	< 0.01	< 0.01
PBF—WHtR	−0.01 (−0.03, −0.01)	−1.40	0.16	0.21	−0.04 (−0.06, −0.01)	−2.85	0.00	0.01
PBF—ABSI	−0.02 (−0.04, 0.01)	−1.50	0.13	0.18	−0.03 (−0.06, 0.01)	−1.38	0.17	0.24
PBF—BRI	−0.04 (−0.05, −0.02)	−4.64	< 0.01	< 0.01	−0.06 (−0.08, −0.03)	−4.78	< 0.01	< 0.01
PBF—AVI	−0.02 (−0.04, −0.01)	−2.49	0.01	0.02	−0.05 (−0.07, −0.03)	−4.91	< 0.01	< 0.01
PBF—CI	−0.04 (−0.06, −0.02)	−4.91	< 0.01	< 0.01	−0.06 (−0.08, −0.03)	−4.15	< 0.01	< 0.01
BMI—HC	0.01 (−0.01, 0.02)	1.17	0.24	0.28	0.03 (0.01, 0.05)	2.66	0.01	0.02
BMI—WC	−0.03 (−0.05, −0.02)	−4.00	< 0.01	< 0.01	−0.02 (−0.04, −0.00)	−2.40	0.02	0.04
BMI—WHR	−0.05 (−0.08, −0.03)	−4.61	< 0.01	< 0.01	−0.04 (−0.07, −0.01)	−2.59	0.01	0.03
BMI—WHtR	−0.02 (−0.04, −0.01)	−2.53	0.01	0.02	−0.01 (−0.03, 0.01)	−0.60	0.55	0.67
BMI—ABSI	−0.02 (−0.06, 0.02)	−1.20	0.23	0.28	0.01 (−0.05, 0.06)	0.21	0.83	0.87
BMI—BRI	−0.04 (−0.06, −0.02)	−4.07	< 0.01	< 0.01	−0.02 (−0.05, 0.00)	−1.94	0.05	0.10
BMI—AVI	−0.03 (−0.04, −0.02)	−3.92	< 0.01	< 0.01	−0.02 (−0.04, −0.00)	−2.36	0.02	0.04
BMI—CI	−0.05 (−0.08, −0.02)	−2.96	0.00	0.01	−0.02 (−0.07, 0.02)	−1.18	0.24	0.31
HC—WC	−0.04 (−0.05, −0.03)	−6.49	< 0.01	< 0.01	−0.05 (−0.07, −0.03)	−4.58	< 0.01	< 0.01
HC—WHR	−0.06 (−0.08, −0.04)	−5.33	< 0.01	< 0.01	−0.07 (−0.11, −0.03)	−3.43	0.01	0.00
HC—WHtR	−0.03 (−0.05, −0.007)	−2.62	0.01	0.02	−0.03 (−0.07, 0.00)	−1.98	0.05	0.09
HC—ABSI	−0.03 (−0.07, −0.01)	−1.67	0.10	0.14	−0.02 (−0.079, 0.03)	−0.78	0.44	0.55
HC—BRI	−0.05 (−0.07, −0.03)	−4.31	< 0.01	< 0.01	−0.05 (−0.09, −0.02)	−2.89	0.00	0.01
HC—AVI	−0.04 (−0.05, −0.03)	−6.47	< 0.01	< 0.01	−0.05 (−0.07, −0.03)	−4.63	< 0.01	< 0.01
HC—CI	−0.05 (−0.08, −0.03)	−3.65	< 0.01	< 0.01	−0.05 (−0.10, −0.01)	−2.30	0.02	0.05
WC—WHR	−0.02 (−0.04, −0.01)	−3.92	< 0.01	< 0.001	−0.017 (−0.035, 0.001)	−1.875	0.061	0.101
WC—WHtR	0.01 (−0.01, 0.02)	1.22	0.22	0.279	0.016 (−0.002, 0.034)	1.703	0.089	0.137
WC—ABSI	0.01 (−0.02, 0.03)	0.45	0.66	0.69	0.03 (−0.01, 0.07)	1.36	0.17	0.24
WC—BRI	−0.01 (−0.03, 0.00)	−1.76	0.08	0.13	−0.00 (−0.02, 0.01)	−0.28	0.78	0.88
WC—AVI	0.00 (0.000, 0.00)	5.06	< 0.01	< 0.01	0.00 (0.000, 0.00)	2.05	0.04	0.08
WC—CI	−0.02 (−0.04, 0.00)	−1.63	0.10	0.15	−0.00 (−0.03, 0.02)	−0.20	0.84	0.86
WHR—WHtR	0.03 (0.02, 0.05)	4.31	< 0.01	< 0.01	0.03 (0.02, 0.05)	3.72	< 0.01	0.00
WHR—ABSI	0.03 (0.01, 0.05)	3.02	0.00	0.01	0.05 (0.02, 0.07)	3.21	0.00	0.00
WHR—BRI	0.01 (0.00, 0.02)	2.94	0.00	0.01	0.02 (0.01, 0.02)	3.42	0.00	0.00
WHR—AVI	0.02 (0.01, 0.04)	3.98	< 0.01	< 0.01	0.02 (−0.00, 0.04)	1.89	0.06	0.10
WHR—CI	0.01 (−0.00, 0.02)	1.47	0.14	0.19	0.01 (0.00, 0.03)	2.01	0.04	0.09
WHtR—ABSI	−0.00 (−0.03, 0.03)	−0.19	0.85	0.85	0.01 (−0.03, 0.05)	0.55	0.58	0.69
WHtR—BRI	−0.02 (−0.03, −0.01)	−4.28	< 0.01	< 0.01	−0.02 (−0.03, −0.01)	−3.45	0.00	0.00
WHtR—AVI	−0.01 (−0.02, 0.01)	−1.11	0.27	0.30	−0.02 (−0.03, 0.00)	−1.60	0.11	0.17
WHtR—CI	−0.03 (−0.05, −0.00)	−2.36	0.02	0.03	−0.02 (−0.05, 0.01)	−1.28	0.20	0.27
ABSI—BRI	−0.02 (−0.04, 0.01)	−1.57	0.12	0.16	−0.03 (−0.06, 0.00)	−1.76	0.08	0.13
ABSI—AVI	−0.01 (−0.03, 0.02)	−0.39	0.70	0.71	−0.03 (−0.07, 0.01)	−1.31	0.19	0.26
ABSI—CI	−0.02 (−0.03, −0.01)	−4.13	< 0.01	< 0.01	−0.03 (−0.05, −0.02)	−3.91	< 0.01	< 0.01
BRI—AVI	0.01 (−0.00, 0.03)	1.86	0.06	0.11	0.00 (−0.01, 0.02)	0.35	0.72	0.84
BRI—CI	−0.00 (−0.02, 0.01)	−0.62	0.53	0.59	0.00 (−0.02, 0.02)	−0.05	0.96	0.96
AVI— CI	−0.02 (−0.04, 0.00)	−1.70	0.09	0.14	−0.00 (−0.03, 0.02)	−0.25	0.80	0.88

P^a^, False Discovery Rate (FDR) were used to adjust p-value in multiple hypothesis testing. ORI, obesity–related indices; AUC, area under curve; CI, confidence interval; PBF, percentage body fat; BMI, body mass index; HC, hip circumference; WC, waist circumference; WHR, waist–hip ratio; WHtR, waist–height ratio; ABSI, a body shape index; BRI, body roundness index; AVI, abdominal volume index; CI, conicity index.

Overall, the results of ROC analyses (Figure 1, Table 3) and multiple non-parametric Z-tests (Table 4) showed that the AUC of WHR was the largest, and the AUC of PBF, BMI, and HC showed lower predictive performance in both men and women.

### The optimal cut-off points of 10 types of ORI for predicting hypertension

According to the ROC curves, we determined the optimal cut-off points of 10 types of ORI for predicting hypertension using the maximum Youden's index (Table 5). The Youden's indices of 10 types of ORI were 0.20–0.40 among men and 0.35–0.45 among women. The optimal cut-off points for identifying hypertension in men and women were as follows: PBF (23.55%, 32.55%), BMI (25.72 kg/m^2^, 23.46 kg/m^2^), HC (97.59 cm, 94.82 cm), WC (90.26 cm, 82.78 cm), WHR (0.91, 0.88), WHtR (0.51, 0.55), ABSI (0.08 m^7/6^/kg^2/3^, 0.08 m^7/6^/kg^2/3^), BRI (4.05, 4.32), AVI (16.31 cm^2^, 13.83 cm^2^), and CI (1.23 m^2/3^/kg^1/2^, 1.27 m^2/3^/kg^1/2^).

**Table 5:** The optimal cut points of 10 types of ORI for predicting hypertension.

**Indices**	**Cut–off point**	**Sensitivity (%)**	**Specificity (%)**	**Youden's index**
**Men (*n =* 1,499)**				
PBF (%)	23.55	63.40	61.80	0.25
BMI (kg/m^2^)	25.72	60.90	61.50	0.22
HC (cm)	97.59	61.30	58.80	0.20
WC (cm)	90.26	63.00	68.00	0.31
WHR	0.91	68.10	65.80	0.40
WHtR	0.51	66.80	60.70	0.28
ABSI (m^7/6^/kg^2/3^)	0.08	63.00	69.20	0.32
BRI	4.05	63.40	70.20	0.34
AVI (cm^2^)	16.31	63.00	67.70	0.31
CI (m^2/3^/kg^1/2^)	1.23	71.50	62.80	0.34
**Women (*n =* 1,302)**				
PBF (%)	32.55	62.50	74.50	0.37
BMI (kg/m^2^)	23.46	75.00	61.60	0.37
HC (cm)	94.82	73.30	61.20	0.35
WC (cm)	82.78	76.70	61.60	0.38
WHR	0.88	75.80	67.90	0.44
WHtR	0.55	60.80	75.80	0.37
ABSI (m^7/6^/kg^2/3^)	0.08	72.50	72.30	0.45
BRI	4.32	65.80	75.80	0.42
AVI (cm^2^)	13.83	75.80	61.90	0.38
CI (m^2/3^/kg^1/2^)	1.27	70.80	74.50	0.45

Youden's Index = (sensitivity + specificity – 1). ORI, obesity–related indices; PBF, percentage body fat; BMI, body mass index; HC, hip circumference; WC, waist circumference; WHR, waist–hip ratio; WHtR, waist–height ratio; ABSI, a body shape index; BRI, body roundness index; AVI, abdominal volume index; CI, conicity index.

### The association of 10 types of ORI and hypertension in multivariate logistic regression models

The associations between 10 types of ORI and hypertension in two conditions (per 1-SD increase and over the optimal cut-off points) were listed in Table 6. In the multivariate logistic regression models, potential confounding factors were age, HR, arteriosclerosis, lifestyle (smoking and exercise status), and CRF. All indices were statistically significant (*P* < 0.05) in the crude models. The OR (per 1-SD increase) values of 10 ORI in men and women were 1.96–2.79 and 2.28–2.94, respectively. In the adjusted model, the OR values of women (1.39–1.93) decreased more than men's (1.51–2.06), indicating that women are more affected by potential confounding factors.

**Table 6:** Crude and adjusted ORs (per 1–SD increase and over the optimal cut–off points) of 10 types of ORI in multivariate logistic regression models.

**Indices**	**Model 1** ^**a**^		**Model 2** ^**b**^	
	**Men (*n =* 1,499)**	**Women (*n =* 1,302)**	**Men (*n =* 1,499)**	**Women (*n =* 1,302)**
	**ORs (95% CI)^a^**	**ORs (95% CI)^a^**	**ORs (95% CI)^b^**	**ORs (95% CI)^b^**
**Per 1–SD increase**				
PBF (%)	2.33 (1.92, 2.84)	2.85 (2.23, 3.66)	1.92 (1.50, 2.45)	1.75 (1.25, 2.45)
BMI (kg/m^2^)	2.18 (1.8, 2.58)	2.43 (2.03, 2.91)	2.06 (1.67, 2.53)	1.90 (1.48, 2.45)
HC (cm)	1.96 (1.69, 2.28)	2.28 (1.89, 2.74)	1.85 (1.53, 2.24)	1.91 (1.48, 2.47)
WC (cm)	2.31 (1.96, 2.72)	2.67 (2.21, 3.22)	1.95 (1.60, 2.39)	1.93 (1.47, 2.51)
WHR	2.79 (2.30, 3.37)	2.94 (2.41, 3.59)	2.06 (1.63, 2.62)	1.85 (1.38, 2.52)
WHtR	2.24 (1.90, 2.63)	2.63 (2.17, 3.18)	1.98 (1.62, 2.43)	1.90 (1.45, 2.49)
ABSI (m^7/6^/kg^2/3^)	2.09 (1.79, 2.46)	2.71 (2.19, 3.37)	1.51 (1.21, 1.88)	1.39 (1.00, 1.94)
BRI	2.36 (2.01, 2.77)	2.39 (2.03, 2.81)	1.92 (1.57, 2.35)	1.70 (1.35, 2.15)
AVI (cm^2^)	2.13 (1.84, 2.47)	2.44 (2.05, 2.90)	1.84 (1.54, 2.21)	1.85 (1.46, 2.34)
CI (m^2/3^/kg^1/2^)	2.34 (1.99, 2.77)	2.81 (2.31, 3.43)	1.79 (1.45, 2.23)	1.70 (1.26, 2.31)
**Over the optimal cut–off points**				
PBF (%)	2.77 (2.07, 3.69)	3.70 (2.50, 5.49)	1.97 (1.43, 2.70)	1.80 (1.05, 3.06)
BMI (kg/m^2^)	2.46 (1.85, 3.27)	4.58 (2.98, 7.03)	2.04 (1.49, 2.79)	2.19 (1.24, 3.84)
HC (cm)	2.25 (1.69, 2.99)	4.29 (2.81, 6.53)	1.80 (1.27, 2.54)	2.18 (1.44, 3.30)
WC (cm)	3.56 (2.66, 4.75)	6.75 (4.50, 10.12)	2.36 (1.72, 3.25)	2.24 (1.41, 3.95)
WHR	3.89 (2.84, 5.32)	6.53 (4.09, 10.43)	2.48 (1.67, 3.67)	2.50 (1.44, 4.36)
WHtR	3.09 (2.30, 4.14)	4.71 (3.20, 6.95)	2.24 (1.63, 3.08)	2.48 (1.55, 3.97)
ABSI (m^7/6^/kg^2/3^)	3.58 (2.67, 4.80)	6.79 (4.35, 10.59)	2.28 (1.55, 3.37)	2.25 (1.24, 3.93)
BRI	4.06 (3.03, 5.44)	5.98 (4.01, 8.92)	2.64 (1.95, 3.56)	2.14 (1.25, 3.67)
AVI (cm^2^)	3.54 (2.65, 4.73)	5.07 (3.28, 7.82)	2.49 (1.91, 3.24)	2.05 (1.25, 3.65)
CI (m^2/3^/kg^1/2^)	4.01 (2.93, 5.50)	6.09 (4.03, 9.21)	2.49 (1.66, 3.74)	2.41 (1.45, 3.97)

a , crude model;

b , adjusted for age;

HR, arteriosclerosis, lifestyle (smoking and exercise status), and CRF; ORI, obesity–related indices; ORs, odds ratios; CI, confidence interval; PBF, percentage body fat; BMI, body mass index; HC, hip circumference; WC, waist circumference; WHR, waist–hip ratio; WHtR, waist–height ratio; ABSI, a body shape index; BRI, body roundness index; AVI, abdominal volume index; CI, conicity index; HR, rest heart rate; CRF, cardiorespiratory fitness. The participants under the optimal cut–off points were taken as the reference, and standardized OR values of above the optimal cut–off points were calculated for each index.

According to the optimal cut-off points of the 10 types of ORI in Table 5, we divided participants into two groups (under and over the optimal cut-off points). Similarly, we analyzed the associations between ORI and hypertension using multivariate logistic regression models. The OR values of 10 ORIs in men and women were 2.25–4.06 and 3.70–6.75, respectively. Women's (1.80–2.50) decreased more than men's (1.80–2.64) after adjusting for the potential confounding factors.

### Sensitivity analysis

When using the hypertension alternative cut-off point (SBP ≥ 135 mmHg and/or DBP ≥ 85 mmHg) to re-run the ROC analysis of the 10 types of ORI, the results showed that the ROC curve, overall model quality plots, and AUC of the 10 types of ORI were roughly similar to the above results (Supplementary material).

## Discussion

Obesity and hypertension, two major risk factors for CVD, contribute to global health and economic burdens (1, 2, 5, 6, 41). Given the dramatic increase in hypertension with the significant rise in obesity, early detection of hypertension using ORI screening could effectively prevent future hypertension and CVD risk. This study adopted ROC analysis to compare the predictive performance of 10 types of ORI for hypertension risk. Generally, the predictive performance is determined by the AUC of the ROC analysis. Some previous studies only used the rank of AUC values to determine predictive performance (4, 5, 8, 25). However, this method was unreliable. The AUC value is an expression of point estimation in statistics, which cannot be considered as a strength or weakness, only referring to the ranked value. Further statistical procedures should be used to compare statistical differences among different AUC values. Therefore, we used the multiple non-parametric Z test to compare differences in AUC among 10 types of ORI for predicting hypertension. Meanwhile, we adjusted the *P*-values in multiple hypothesis testing by FDR. Based on a cross-sectional study of 2,801 participants, the results showed that the 10 types of ORI (PBF, BMI, HC, WC, WHR, WHtR, ABSI, BRI, AVI, and CI) could predict hypertension, among which WHR should be recommended as the best predictor. We believe those results may help the Chinese population to select the appropriate ORI to estimate the risk of hypertension.

The multiple non-parametric Z test results showed that central ORI (WC, WHR, and AVI) had a better predictive performance for identifying hypertension compared to general ORI (PBF and BMI). Some studies showed that CVD risk factors were more strongly associated with central obesity than with general obesity in Chinese (30), Japanese (49), Thai (50), and Indian (51, 52), which was in agreement with our findings for hypertension. A Brazilian survey showed that women with abdominal obesity were 30% more likely to develop hypertension than those with general obesity (53). One reason for the strong association between central obesity and hypertension may be that excess abdominal fat would lead to increased insulin resistance (54). Regarding obesity-related hypertension, insulin resistance may synergistically affect the obesity–hypertension association by increasing adipokine secretion and sympathetic nervous system activity (55, 56). Insulin resistance may induce renal sodium retention, activate the renin–angiotensin system and enhance the sympathetic nervous system activity, and promote endothelial dysfunction, and increase peripheral and renal vascular resistance (57, 58). However, the precise pathophysiological mechanisms contributing to the development of obesity-related hypertension have not been elucidated (59). Therefore, further studies are needed to investigate the causal relationship between hypertension and obesity.

The prevention of hypertension cannot be limited only to obese people. Recently, CRF has been advocated as a risk factor for CVD, given its strong inverse association with adverse outcomes, particularly all-cause mortality, and cardiovascular events (39). Ge et al. showed that the optimal ORI for predicting hypertension differed among women in different age groups (5). Previous studies have shown that factors such as sex, arteriosclerosis, and lifestyle may influence obesity and hypertension (5, 38). Therefore, when analyzing the association between ORI and hypertension, potential confounding factors were age, HR, arteriosclerosis, lifestyle (smoking and exercise status), and CRF in this study. When there are multiple potential confounders, multivariate logistic regression model has the advantage of controlling the mixed relationship among the multiple factors, which is a better statistical method (38). Therefore, we used a multivariate logistic regression model to control the confounding factors for analyzing the association between ORI and hypertension in two conditions (per 1-SD increase and over the optimal cut-off points). The units of each index in this study were inconsistent, so standardized indices were used in the models. In the crude models, multivariate logistic regression model results demonstrated that the OR values of all ORI of women were higher than those of men in the two conditions. However, women's OR values decreased more than those of men after adjusting for potential confounding factors, and even the ORs of some indices were smaller than those of men. This could be partially explained by women being perhaps more affected by potential confounding factors. Further study needs to be explored concerning the effects of those potential confounding factors on hypertension.

This study has determined the optimal cut-off points of 10 types of ORI to predict hypertension by the maximum Youden's index. According to the literature (60, 61), the Working Group on Obesity in China (WGOC) also established BMI and WC cut-off points for Chinese adult obesity criteria. The optimal cut-off points for BMI identified in this study (25.72 among men and 23.46 kg/m^2^ among women) were lower than obesity (28 kg/m^2^) as defined by the WGOC but close to the WGOC cut-off point of 24 kg/m^2^ for overweight (60, 61). In addition, WGOC indicated that being overweight had better sensitivity and specificity for identifying risk factors. Accordingly, the overweight criteria by BMI can also be used to predict hypertension appropriately. The WC optimal cut-off points for predicting hypertension were 90.26 (men) and 82.78 cm (women), respectively. Since the WC cut-off point for obesity in Chinese adults established by WGOC (85 cm among men and 80 cm among women) is mainly determined based on the comprehensive CVD risk, it may not be suitable when predicting hypertension alone. However, the cut-off points of PBF for Chinese adult obesity criteria have yet to be developed due to a lack of national data on PBF and CVD. In this study, the PBF optimal cut-off points were 23.55% among men and 32.55% among women for predicting hypertension, which is close to the World Health Organization's (WHO) definition of obesity by PBF (≥ 25% for men and ≥ 35% for women) (62). Although PBF reflected body composition more accurately than traditional ORI, in this study, PBF had merely no advantage in predicting hypertension compared with central ORI (WC, WHR, and AVI). Therefore, our findings indicated that the optimal cut-off points of central ORI may be more significant in identifying hypertension risk for adults in China.

These 10 types of ORI and the optimal cut-off points of these indices were suitable for predicting hypertension in the Chinese population. However, it does not mean that they can be effectively applied to predict other CVDs or hypertension in other regions. For example, a study exploring the association between ORI and diabetes showed that BMI and WC are better indices for diabetes screening (63). A Singapore study indicated that integrating BMI and WHtR has better clinical utility in evaluating CVD risk factors (8). In addition, a study of Iranian adults suggested that ABSI is a weak predictor of CVD risks (64). Hence, the predictive performance of the 10 types of ORI for identifying other CVDs or hypertension in other regions need to be studied further.

### Limitations

Regarding limitations, this study did not provide a causal relationship between ORI and hypertension because it was a cross-sectional study. Further studies are needed to validate the optimal cut-off point. Next, covariates such as smoking and exercise are based on self-reports. Other variables that might influence hypertension were not collected, such as drinking alcohol; the results may therefore be less reliable. Finally, the participants were predominately from Ningbo city and could not represent the whole Chinese population.

## Conclusions

All 10 types of ORI (PBF, BMI, HC, WC, WHR, WHtR, ABSI, BRI, AVI, and CI) can effectively predict hypertension, among which WHR should be recommended as the best predictor. The optimal cut-off points for the 10 ORI for predicting hypertension were determined. Moreover, central ORI (WC, WHR, and AVI) have better prediction performance than general ORI (PBF and BMI) when predicting the risk of hypertension. Therefore, greater emphasis should be placed on the measurement of central ORI in future studies surveying the relationship between risk factors for hypertension in the Chinese population.

## Data availability statement

The original contributions presented in the study are included in the article/Supplementary material, further inquiries can be directed to the corresponding author.

## Ethics statement

The studies involving human participants were reviewed and approved by Design and protocol of this study were approved by the Institutional Review Board of the Faculty of Sports Science, Ningbo University (NO. 2018RAGH1025). The patients/participants provided their written informed consent to participate in this study.

## Author contributions

XL and HH conceived the presented idea, developed the framework, and wrote the manuscript. YG, YF, YZ, and LG were involved in the data collection. YJ, AW, and HH provided critical feedback and contributed to the final version. All authors have read and agreed to the published version of the manuscript.

## Funding

This research was funded by the National Social Science Foundation of China (Grant number 18BTY100).

## Conflict of interest

The authors declare that the research was conducted in the absence of any commercial or financial relationships that could be construed as a potential conflict of interest.

## Publisher's note

All claims expressed in this article are solely those of the authors and do not necessarily represent those of their affiliated organizations, or those of the publisher, the editors and the reviewers. Any product that may be evaluated in this article, or claim that may be made by its manufacturer, is not guaranteed or endorsed by the publisher.

## Abbreviations

ABSI: a body shape index
AUC: areas under the curve
AVI: abdominal volume index
baPWV: brachial–ankle pulse wave velocity
BMI: body mass index
BRI: body roundness index
CI: conicity index
CRF: cardiorespiratory fitness
CVAI: Chinese visceral adiposity index
CVD: cardiovascular disease
DBP: diastolic blood pressure
FDR: False Discovery Rate
HC: hip circumference
HDL–C: high density lipoprotein–cholesterol
HR: rest heart rate
LAP: lipid accumulation product
METs: metabolic equivalent values
ORI: obesity–related indices
ORs: odds ratios
PBF: percentage body fat
ROC: receiver operator characteristic
SBP: systolic blood pressure
SD: standard deviation
STROBE: strengthening the reporting of observational studies in ipidemiology
TG: triglyceride
WC: waist circumference
WGOC: Working Group on Obesity in China
WHO: World Health Organization
WHR: waist–hip ratio
WHtR: waist–height ratio.
