# Supplementary material for: Comparison of 10 obesity-related indices for predicting hypertension based on ROC analysis in Chinese adults
Source: Front Public Health. 2022 Nov 25;10:1042236. doi: 10.3389/fpubh.2022.1042236 (PMC9732655; doi:10.3389/fpubh.2022.1042236)
Supplement: Supplementary file 1 [file Table_1.DOCX]

Supplementary Material

**Supplementary Table 1.** The AUC of ten ORI for predicting hypertension.

| **Indices** | **Men (n = 1,499)** |  | **Women (n = 1,302)** |  |
| --- | --- | --- | --- | --- |
|  | **AUC (95% CI)** | ***p*** | **AUC (95% CI)** | ***p*** |
| PBF (%) | 0.70 (0.67, 0.73) | < 0.01 | 0.73 (0.69, 0.77) | < 0.01 |
| BMI (kg/m^2^) | 0.70 (0.67, 0.73) | < 0.01 | 0.76 (0.72, 0.80) | < 0.01 |
| HC (cm) | 0.69 (0.66, 0.72) | < 0.01 | 0.73 (0.69, 0.77) | < 0.01 |
| WC (cm) | 0.73 (0.70, 0.75) | < 0.01 | 0.78 (0.75, 0.82) | < 0.01 |
| WHR | 0.74 (0.72, 0.77) | < 0.01 | 0.80 (0.76, 0.83) | < 0.01 |
| WHtR | 0.71 (0.69, 0.74) | < 0.01 | 0.77 (0.73, 0.80) | < 0.01 |
| ABSI (m^7/6^/kg^2/3^) | 0.71 (0.68, 0.73) | < 0.01 | 0.75 (0.70, 0.79) | < 0.01 |
| BRI | 0.73 (0.71, 0.76) | < 0.01 | 0.78 (0.75, 0.82) | < 0.01 |
| AVI (cm^2^) | 0.72 (0.70, 0.75) | < 0.01 | 0.78 (0.74, 0.82) | < 0.01 |
| CI (m^2/3^/kg^1/2^) | 0.73 (0.70, 0.76) | < 0.01 | 0.78 (0.74, 0.82) | < 0.01 |

Abbreviations: ORI, obesity-related indices; PBF, percent body fat; BMI, body mass index; HC, hip circumference; WC, waist circumference; WHR, waist–hip ratio; WHtR, waist–height ratio; ABSI, a body shape index; BRI, body roundness index; AVI, abdominal volume index; CI, conicity index.

**Supplementary Figure 1.** ROC analysis of ten ORI for predicting hypertension. A, men’s ROC curve; B, women’s ROC curve; C, men’s overall model quality; D, women’s overall model quality. Abbreviations: ORI, obesity-related indices; PBF, percent body fat; BMI, body mass index; HC, hip circumference; WC, waist circumference; WHR, waist–hip ratio; WHtR, waist–height ratio; ABSI, a body shape index; BRI, body roundness index; AVI, abdominal volume index; CI, conicity index.
